# Induction of cell proliferation in old rat liver can reset certain gene expression levels characteristic of old liver to those associated with young liver

**DOI:** 10.1007/s11357-012-9404-z

**Published:** 2012-04-04

**Authors:** Muhammad A. Chishti, Namik Kaya, Al-Bandary BinBakheet, Falah Al-Mohanna, Malcolm H. Goyns, Dilek Colak

**Affiliations:** 1 Department of Comparative Medicine, King Faisal Specialist Hospital and Research Centre, Riyadh, Saudi Arabia 11211; 2 Department of Genetics, King Faisal Specialist Hospital and Research Centre, Riyadh, Saudi Arabia 11211; 3 Children’s Cancer Center, King Faisal Specialist Hospital and Research Centre, Riyadh, Saudi Arabia 11211; 4 Department of Biostatistics, Epidemiology and Scientific Computing, King Faisal Specialist Hospital and Research Centre, Riyadh, Saudi Arabia 11211; 5 Present Address: Department of Pathology, Clinical Biochemistry Unit, Obesity Research Center, College of Medicine, King Saud University, Riyadh, Saudi Arabia 11461; 6 Present Address: Immorgene Concepts Ltd., Stockton-on-Tees, TS22 5YA UK

**Keywords:** Liver, Aging, Hepatectomy, Microarray, Global gene expression, Regeneration, Longevity

## Abstract

During the past decade, it has become increasingly clear that consistent changes in the levels of expression of a small cohort of genes accompany the aging of mammalian tissues. In many cases, these changes have been shown to generate features that are characteristic of the senescent phenotype. Previously, a small pilot study indicated that some of these changes might be reversed in rat liver, if the liver cells became malignant and were proliferating. The present study has tested the hypothesis that inducing proliferation in old rat liver can reset the levels of expression of these age-related genes to that observed in young tissue. A microarray approach was used to identify genes that exhibited the greatest changes in their expression during aging. The levels of expression of these markers were then examined in transcriptomes of both proliferating hepatomas from old animals and old rat liver lobes that had regenerated after partial hepatectomy but were again quiescent. We have found evidence that over 20 % of the aging-related genes had their levels of expression reset to young levels by stimulating proliferation, even in cells that had undergone a limited number of cell cycles and then become quiescent again. Moreover, our network analysis indicated alterations in MAPK/ERK and Jun-N-terminal kinase pathways and the potential important role of *PAX3*, *VCAN*, *ARRB2*, *NR1H2*, and *ITGA5* that may provide insights into mechanisms involved in longevity and regeneration that are distinct from cancer.

## Introduction

It has become increasingly clear that changes in the expression levels of certain genes accompany the aging of mammals. Over a decade ago, studies using the differential display technique demonstrated that approximately 1 % of all active genes exhibit such age-related changes, some increasing during aging and others decreasing (Goyns et al. 1998; Salehi et al. 1996). An important observation from these early studies was that the genes involved were consistent from individual to individual and in some cases from species to species (Goyns 2002). The advent of microarray technology allowed these general conclusions to be confirmed, but also provided a far more rapid identification of the genes exhibiting these age-related changes. Most importantly, the microarray studies demonstrated that the changes in gene expression were delayed in animals that had been subjected to calorie restriction diets (Lee et al. 1999, 2000; Li et al. 1997; Kume et al. 2010). As a calorie restriction diet is the only experimental procedure that can consistently extend life span in mammals (Masoro 1992), these studies lend support to the hypothesis that changes in gene expression levels are important in the development of the senescent phenotype (Swindell 2008).

More recently, changes in gene expression have been reported in a wide range of tissues, including brain (Lu et al. 2004), kidney (Rodwell et al. 2004; Kume et al. 2010), liver (Cao et al. 2001; Mori et al. 2007), fibroblasts(Ly et al. 2000), cardiac muscle (Lee et al. 2002), and skeletal muscle (Welle et al. 2003). These studies have studied species as diverse as mice (Cao et al. 2001; Lee et al. 2002), monkeys (Kayo et al. 2001), and humans (Welle et al. 2003). Increasingly, these gene expression changes are being linked to physiological aging (Rodwell et al. 2004; Zahn et al. 2006).

It is intriguing to question whether it is possible to develop an intervention that could have an anti-aging effect. The application of calorie restriction diets in mice has already demonstrated that a general slowing of these gene expression changes can be achieved (Cao et al. 2001; Lee et al. 1999). Also, a small pilot study has reported that some of the age-related changes in gene expression observed in rat liver appear to be reversed in hepatoma cells derived from old animals (Charlton et al. 1999; Tanaka et al. 2002). In the latter study, it was not clear whether the reversal effect was related to the hepatoma cells being malignant or whether it was a result of the cells actively proliferating.

In present study, we investigated the hypothesis that stimulation of cell proliferation in old livers can reset age-related gene expression levels to those observed in young liver using a microarray approach. We analyzed the transcriptomes of proliferating hepatoma cells, regenerated old liver, and normal aging liver in a rat model using a microarray of more than 27,000 annotated genes from Celera and public repositories. Hence, we explored possible molecular links between regeneration, cancer, and longevity: common genes, networks, and pathways associated with aging, regeneration, and cancer.

## Materials and methods

### Animals

A colony of Sprague Dawley rats was maintained at the King Fahad National Centre for Children's Cancer and Research animal house, which is managed in accordance with AALAS regulations. A total of 60 rats, including 30 young rats at the age of 5 months and 30 old rats at the age of 22 months were used in this study. These young rats were grouped as normal young (denoted as NY), drug-induced hepatoma (DY), and regenerating liver by partial hepatectomy (RY), each consisting of 10 rats. Similarly, 30 old rats were grouped as normal old (NO), drug-induced hepatoma (DO), and regenerating liver by partial hepatectomy (RO), each consisting of 10 rats.

Ten rats from the young and old age groups were subjected to partial hepatectomy. Briefly, a midline ventral abdominal skin incision was made, and a small bolster was placed under the thorax causing the liver to fall slightly forward away from the diaphragm; then, the ligaments attaching the liver to the diaphragm were cut down. A piece of gauze was placed near the incision, and the left lateral and median lobes were moved out from the abdominal cavity. These lobes were lifted vertically, blood vessels were tied then laid on the gauze, and a cut was made to bleed on the gauze, not into the abdominal cavity, and finally transected. The bolster was removed, and the skin incisions were closed (Wayneforth 1980). The remaining liver lobes were allowed to undergo the regeneration process, which was completed within 1 month, by which time the liver cells had again become quiescent. This was confirmed by a histological analysis of sections from the liver. The rats were sacrificed after 1 month of partial hepatectomy, and samples were collected from four rats in each group.

In parallel, young and old rats (10 each) were treated with diethylnitrosoamine (200 mg/kg), which was injected intraperitoneally to induce the formation of a hepatoma (Ito et al. 1989). Tumor formation became apparent within 4 weeks. The rats were sacrificed, and samples were collected. All tissues were snap frozen and stored at –80 °C until required for RNA isolation. Small pieces of tissue were also removed from the regenerated lobes and hepatoma foci at the same time, subjected to formalin fixation, and examined histologically after hematoxylin and eosin staining. Samples were collected from a minimum of four rats in each group. We used four individual animals in each group (DO, DY, RO, RY, NO, and NY) for further study.

### RNA isolation

RNA was prepared from the rat tissue samples using the Trizol protocol (Invitrogen, Carlsbad, CA, USA). Total RNA quality and quantities were determined by measuring the absorbance spectra on a UV/vis spectrophotometer, the NanoDrop® ND-1000 Spectrophotometer (Nanodrop Inc., Wilmington, DE, USA), and further analyzed by an RNA 6000 Nano Assay using the 2100 Bioanalyzer (Agilent Technologies, Santa Clara, USA). The undegraded, higher-quality RNA was processed further for RT-PCR and microarray experiments.

### Microarray hyridization

Whole-genome gene expression profiling of 24 samples from hepatoma cells (DO, DY; *n* = 8), regenerated liver (RO, RY; *n* = 8), and normal liver (NO, NY; *n* = 8) using Applied Biosystems Rat Genome Survey Microarray (Applied Biosystems, Foster City, CA, USA) contains more than 27,000 gene sequences based on 60-mer oligonucleotide probes. Digoxigenin-UTP-labeled cRNA was generated and amplified from 2 μg total RNA from each sample using applied Biosystems Chemiluminescent RT-IVT labeling kit v 1.0 (Applied Biosystems, Foster City, CA, USA) following the manufacturer's protocol. Array hybridization was performed for 16 h at 55 °C. Chemiluminescence detection, image acquisition, and analysis were performed using Applied Biosystems Chemiluminescence Detection Kit and the Applied Biosystems 1700 Chemiluminescent Microarray Analyzer (Applied Biosystems, Foster City, CA, USA), following the manufacturer's protocol.

### Microarray data analysis

Images were auto-gridded, the chemiluminescent signals were quantified, and the background subtracted using the Applied Biosystems 1700 Chemiluminescent Microarray Analyzer software v 1.1. For transcriptome analysis, detection thresholds were used following the manufacturer's recommendations. Detection threshold was set as S/N >3 and quality flag <5,000. Various Bioconductor packages were used for normalization and determination of differentially expressed genes (Gentleman et al. 2004). Significantly modulated genes were defined as those with absolute fold change >2.0 and an analysis of variance (ANOVA) *p* value <0.05. Human hepatocellular carcinoma (HCC) datasets from independent studies were analyzed as described previously (Colak et al. 2010). The hierarchical clustering of differentially expressed genes using Pearson's correlation with average linkage clustering was performed using the TIGR Multi Experiment Viewer (Saeed et al. 2003), and heatmaps were generated with red and green indicating high and low expression, respectively.

Functional annotation and biological term enrichment analysis was performed by using the protein analysis through evolutionary relationships (PANTHER) classification system (Thomas et al. 2003). For each molecular function, biological process, or pathway term, PANTHER calculates the number of genes identified in that category in both the list of differentially regulated genes and a reference list containing all the probe sets present on the AB Human Genome Survey Microarray and compares these results using the binomial test to determine if there are more genes than expected in the differentially regulated list (Thomas et al. 2006). Over-representation was defined by a *p* value <0.05. Functional pathway and gene interaction network analyses were executed using Ingenuity Pathways Analysis (IPA) 6.3 (Ingenuity Systems, Mountain View, CA). Statistical analyses were performed with the MATLAB software packages (Mathworks, Natick, MA, USA), R/Bioconductor, and PARTEK Genomics Suite (Partek Inc., St. Louis, MO, USA).

### Real-time RT-PCR

In order to validate our microarray results, confirmatory real-time RT-PCR was performed using the ABI 7500 Sequence Detection System (ABI, Foster City, CA, USA). For this purpose, 50 ng total RNA procured from the same microarray study samples was transcribed into cDNA using Sensiscript Kit (QIAGEN Inc., Valencia, CA, USA) according to the manufacturer's recommendations. Eight differentially expressed genes were randomly selected and primers designed using Primer3 software (Table 1). After primer optimization, the PCR assays were performed in 6 μl of the cDNA using the QIAGEN QuantIT SyBR Green Kit, employing GAPDH as the endogenous control gene. All reactions were conducted in triplicates, and the data were analyzed using the delta delta C_T_ method (Livak and Schmittgen 2001).

**Table 1:** Nucleotide sequences used in real-time RT-PCR validation of randomly selected genes identified by microarray analysis

Gene	Forward primer	Reverse primer
Ube2l6	GGTGAGAAGGCAGGACTCTG	TTTTGTGAGTCATCAACAGAAAAT
Wit3	TCCAGCAAGAAAACAGACAAA	TCCTTTGAGTGCTGCTCCTT
Caskin1	TACTACATCCCAGGCCAGCA	TGGATGCTGTTCAAGTACCG
Cltb	GGAACCTGCAACCTGTCTGT	CGAGAAAGCTAAGGTTCCCC
Esm1	TTGCCTCCTGAGAAACAGAA	GGTTCTCAAACACTCCTACATGG
Nrp1	AACTGGTCTGGATGGTGGTC	AACCACATTCCTCAGGAGGA
Cap350	ACACCCCGTGCAGCTCTTAG	GAAGTCCCATGTATACCCTGTAAA
E2F5	TTGACCAGCAGAAGTTGTGG	ATTCAGGCACCCTCTGGTAC

## Results

### Global gene expression profiling of normal aging liver, hepatoma, and regenerated old liver

**Fig. 1:**
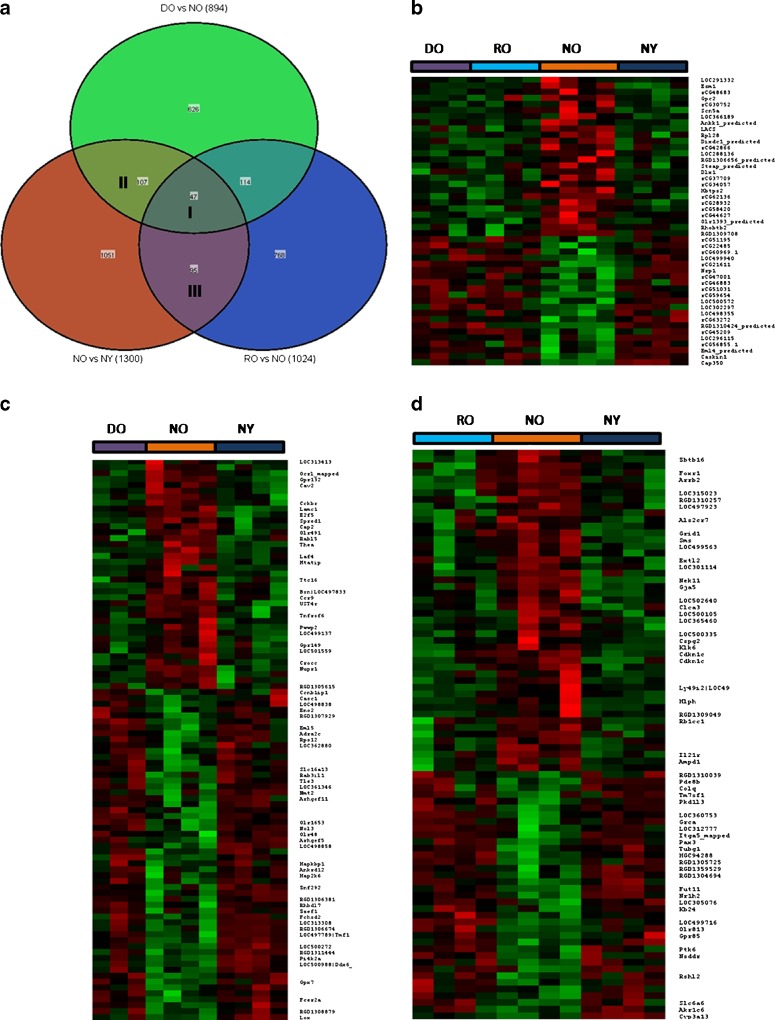
**a** Venn diagram indicating the significantly regulated gene overlaps for three comparisons. **b**–**d** Heatmaps of genes in the intersection I, II, and III indicated in the Venn diagram (Fig. 1**a**), respectively. Gene symbols are listed on the *right*. In heatmaps, *rows* represent genes, and *columns* represent samples from DO (hepatoma in old), RO (regeneration in old), NO (normal old), and NY (normal young) groups. Genes are clustered using row normalized signals and mapped to the [−3,3] interval. *Red* and *green* represent high and low expression values, respectively

We analyzed whole-genome mRNA expression profiling of hepatoma cells, regenerated liver from old rats, and normal liver of both young and old rats using Applied Biosystems Rat Genome Survey microarray which includes more than 27,000 annotated genes. First, we indentified normal aging signature genes by comparing the transcriptomes of normal young and old livers. The ANOVA identified 1,300 probes (corresponding to 397 up- and 519 downregulated genes) as differentially expressed in normal old compared to normal young (*p* value <0.05 and absolute fold change of >2.0). The levels of expression of these genes were then compared to the hepatomas derived from old liver and the regenerated old liver by using overlapping gene lists (Fig. 1a). When comparing two groups of samples to identify genes differentially expressed in a given group, we used *p* value and the fold change (FC) between two groups as the cutoff criteria. If the *p* value is <0.05 and the absolute FC between the groups is >2.0, the corresponding gene was considered differentially expressed between the two groups. Each circle in the Venn diagram represents the differential expression between two “treatment” types (Fig. 1a). The red circle (left) shows the 1,300 normal aging genes that are differentially expressed between NO and NY; 142 and 154 of those genes were also differentially expressed in regenerated liver (RO) and hepatomas from old liver, respectively, 47 of which were common to all comparisons (Fig. 1a, listed in Table 2). It is clearly seen from the heatmaps that over 90 % of genes commonly dysregulated in aging and hepatoma as well as in regeneration have similar levels of expression as in normal young liver (NY), but not from normal old liver (NO) (Fig. 1b–d), where red indicates high levels of expression and green indicates low levels of expression. Intriguingly, we identified 95 aging genes that are significantly dysregulated in the regenerated old liver; however, their expressions in the regenerated old liver were reversed (Fig. 1a, region III, and Fig. 1d). This excludes the 47 aging genes that were also dysregulated in hepatomas from old liver (Fig. 1a, region I). Indeed, as in the hepatoma, expression levels of over 90 % of aging genes in the regenerated old liver were also reset to those seen in the normal young liver (Fig. 1d). The heatmap of those genes were shown in Fig. 1d and listed in Table 3. Importantly, this set of genes corresponds to ones that are exclusively due to cells that had undergone proliferation (in old liver), but not due to cells undergoing malignancy (hepatoma) and have similar level of expression to those seen in the normal young liver.

**Table 2:** Selected differentially up- or downregulated genes in both regenerated (RO) liver and hepatomas (DO) from old liver whose expression levels were also reset to those seen in normal young (NY) liver (Fig. 1a, region I)

Gene	Gene name	NY/NO^a^	DO/NO^b^	RO/NO^b^
Dixdc1	DIX domain containing 1	−2.9	−2.7	−3.4
Ankk1	Ankyrin repeat and kinase domain containing 1	−3.0	−2.3	−3.2
Esm1	Endothelial cell-specific molecule 1	−5.2	−3.4	−3.2
Gpc2	Glypican 2	−3.7	−3.6	−3.1
Rhobtb2	Rho-related BTB domain containing 2	−2.1	−2.8	−2.8
Rpl28	Ribosomal protein L28	−2.9	−3.3	−2.7
Dlx1	Distal-less homeobox 1	−2.7	−2.4	−2.6
Mbtps2	Membrane-bound transcription factor peptidase, site 2	−2.5	−2.2	−2.6
LACS	L-NAME induced actin cytoskeletal protein	−3.0	−2.6	−2.5
RGD1565996	Similar to DnaJ homolog subfamily B member 6 (Heat shock protein J2)	−2.8	−3.0	−2.5
Tgm3	Transglutaminase 3, E polypeptide	−3.1	−3.5	−2.5
Olr1393	Olfactory receptor 1393	−2.1	−2.2	−2.4
Scn5a	Sodium channel, voltage-gated, type V, alpha polypeptide	−3.3	−2.3	−2.1
Eml4	Echinoderm microtubule associated protein like 4	3.6	2.7	2.2
Nrp1	Neuropilin 1	2.4	2.5	2.4
RGD1562100	Similar to ADP-ribosylation factor interacting protein 2 (arfaptin 2)	3.0	3.1	3.1
Caskin1	CASK-interacting protein 1	3.6	3.4	3.4
Cap350	Centrosome-associated protein 350	8.1	3.5	3.4

^a^Fold change (FC) was calculated between the mean values of NO (normal old) and NY (normal young)

^b^Fold change (FC) was calculated between the mean values of NO (normal old) and DO (hepatoma) as well as RO (regenerated)

**Table 3:** Top 38 genes that were most significantly dysregulated in regenerated old liver (excluding genes that are shared in hepatomas from old liver) whose expressions were reset to the normal young status (Fig. 1a, region III)

Gene symbol	Gene name	Biological process	FC NY/NO^a^	FC RO/NO^b^
Cspg2	Chondroitin sulfate proteoglycan 2	Cell motility | signal transduction | extracellular matrix protein-mediated signaling; cell proliferation and differentiation; cell structure and motility | cell communication	−5.5	−5.6
Grid1	Glutamate receptor, ionotropic, delta 1	Synaptic transmission | transport | neuronal activities	−18.9	−4.8
Tll2	Tolloid-like 2	Proteolysis; signal transduction | protein metabolism and modification | ligand-mediated signaling; developmental processes | mesoderm development | cell communication | skeletal development	−3.6	−3.1
Zbtb16	Zinc finger and BTB domain containing 16	Nucleoside, nucleotide and nucleic acid metabolism | mRNA transcription	−2.4	−2.9
RGD1310257	Similar to RIKEN cDNA 6330408A02 gene	Protein metabolism and modification | proteolysis; developmental processes | neurogenesis; cell proliferation and differentiation | ectoderm development	−2.6	−2.9
Arrb2	Arrestin, beta 2	Cell surface receptor-mediated signal transduction | signal transduction | G protein-mediated signaling; intracellular protein traffic | endocytosis; sensory perception	−2.6	−2.6
Cdkn1c	Cyclin-dependent kinase inhibitor 1C	Tumor suppressor | cell cycle | cell cycle control; cell proliferation and differentiation; oncogenesis	−3.2	−2.6
Extl2	Exotoses (multiple)-like 2	Other polysaccharide metabolism; protein metabolism and modification | protein glycosylation | carbohydrate metabolism	−3.8	−2.6
Slc25a32	Solute carrier family 25, member 32	Small molecule transport | transport	−2.2	−2.5
Foxr1	Forkhead box R1	Tissue development; embryo development; organ development	−2.4	−2.5
Ampd1	Adenosine monophosphate deaminase 1 (isoform M)	Nucleoside, nucleotide, and nucleic acid metabolism | purine metabolism	−2.1	−2.5
RGD1309049	Similar to RIKEN cDNA 4933415F23	Protein phosphorylation; protein targeting and localization | protein metabolism and modification | protein targeting | protein modification	−2.2	−2.4
Slc25a41	Solute carrier family 25, member 41	Small molecule transport | transport	−2.3	−2.4
LOC500105	Similar to contactin associated protein-like 2 isoform a	Synaptic transmission | signal transduction | cell communication | cell adhesion-mediated signaling; cell adhesion; neuronal activities	−2.3	−2.3
Mlph	Melanophilin	General vesicle transport | intracellular protein traffic	−3.0	−2.3
Nek11	NIMA (never in mitosis gene a)-related kinase 11	Protein metabolism and modification | protein modification | protein phosphorylation; phosphate metabolism; other metabolism	−4.5	−2.2
Cdkn1c	Cyclin-dependent kinase inhibitor 1C (P57)	Tumor suppressor | cell cycle | cell cycle control; cell proliferation and differentiation; oncogenesis	−2.9	−2.2
Klk6	Kallikrein 6	Proteolysis | protein metabolism and modification	−2.2	−2.1
Gja5	Gap junction membrane channel protein alpha 5	Signal transduction	−2.6	−2.1
Il21r	Interleukin 21 receptor	Natural killer cell-mediated immunity | immunity and defense	−2.4	−2.1
Clca3	Chloride channel calcium activated 3	ATP synthesis coupled proton transport	−2.3	−2.1
Akr1c6	Aldo-keto reductase family 1, member C6	Steroid hormone metabolism | steroid metabolism | fatty acid biosynthesis; lipid, fatty acid, and steroid metabolism | fatty acid metabolism | lipid, fatty acid, and steroid metabolism	2.8	2.1
Rshl2	Radial spokehead-like 2	Signal transduction; intracellular protein traffic; protein targeting and localization; miscellaneous	2.1	2.3
Pde8b	Phosphodiesterase 8B	Nucleoside, nucleotide and nucleic acid metabolism | metabolism of cyclic nucleotides; signal transduction	2.7	2.3
Cyp3a13	Cytochrome P450, family 3, subfamily a, polypeptide 13	Steroid hormone metabolism; electron transport | steroid metabolism | lipid, fatty acid, and steroid metabolism	30.2	2.3
Tubg1	Tubulin, gamma 1	Cell motility | chromosome segregation; cell structure and motility | cell structure; cell structure and motility | mitosis | intracellular protein traffic; cell cycle	2.5	2.5
Kb24	Type II keratin Kb24	Ectoderm development; cell structure and motility | cell structure | developmental processes	2.2	2.5
Pax3	Paired box gene 3	Nucleoside, nucleotide, and nucleic acid metabolism | mRNA transcription regulation; developmental processes | segment specification; neurogenesis | ectoderm development	2.4	2.5
Gpr85	G protein-coupled receptor 85	Cell surface receptor-mediated signal transduction | signal transduction | G protein-mediated signaling	2.7	2.7
Fut11	Fucosyltransferase 11	Protein metabolism and modification | protein glycosylation | protein modification	3.8	2.7
Pnpla6	Patatin-like phospholipase domain containing 6	Lipid, fatty acid, and steroid metabolism | other neuronal activity | phospholipid metabolism; neuronal activities	2.1	2.7
Nsddr	Neural stem cell-derived dendrite regulator	Biological process unclassified	2.3	3.0
Nr1h2	Nuclear receptor subfamily 1, group H, member 2	Transcription | regulation of transcription, DNA-dependent | negative regulation of transcription | cellular lipid metabolic process | positive regulation of transcription, DNA-dependent | retinoic acid receptor signaling pathway	3.9	3.1
Ptk6	PTK6 protein tyrosine kinase 6	Protein phosphorylation; signal transduction | protein metabolism and modification | protein modification | intracellular signaling cascade; oncogenesis	2.7	3.3
Itga5	Integrin alpha 5	Cell adhesion	3.0	3.4
Olr813	Olfactory receptor 813	Cell surface receptor-mediated signal transduction | signal transduction | G protein-mediated signaling; sensory perception | olfaction | chemosensory perception	2.3	3.8
Slc6a6	Solute carrier family 6	Transport | small molecule transport; transport | extracellular transport and import	3.9	5.0

^a^Fold change (FC) was calculated between the mean values of NO (normal old) and NY (normal young)

^b^Fold change (FC) was calculated between the mean values of NO (normal old) and DO (hepatoma) as well as RO (regenerated)

### Gene ontology analyses of aging and regenerated old liver transcriptome

To delineate which biological processes are significantly overrepresented in normal aging and regenerated signature genes, we performed gene ontology and biological term enrichment analyses using both the PANTHER classification system and the Ingenuity Pathway Analysis (IPA). Intriguingly, genes related to immune response, cell adhesion, nervous system and development, and response to stress were found to be activated with aging. On the other hand, genes involved with lipid metabolism, cellular growth and maintenance, protein synthesis, and cellular communication were downregulated. The regeneration-specific genes, however, were mainly associated with lipid metabolism, mRNA transcription and regulation, protein modification, protein phosphorylation, cell morphology, cellular development, small molecule biochemistry, and cellular growth and proliferation. Thus, the genes and processes activated or repressed by aging were conversely regulated in the regenerated liver.

**Fig. 2:**
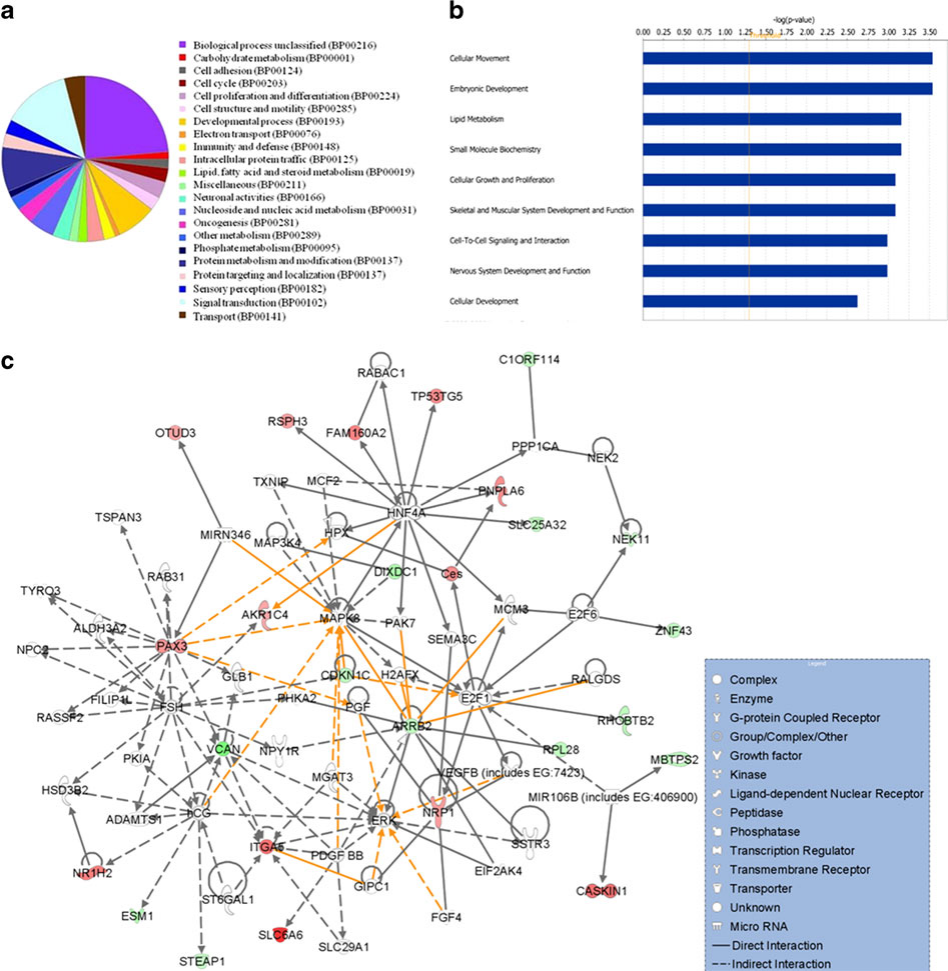
Functional and network analyses of aging and regenerated liver transcriptome. PANTHER pie chart of biological processes (**a**), significantly enriched functional categories (**b**), and gene interaction network (**c**) analysis of regeneration-specific up/downregulated genes that are also common to normal aging genes, and whose expressions were reset to the normal young status. **b** *X-axis* indicates the significance (-log *p* value) of the functional association that is dependent on the number of genes in a class as well as biologic relevance. **c** *Nodes* represent genes, with their shape representing the functional class of the gene product, and *edges* indicate biological relationship between the nodes (see *legend*). *Green* indicates downregulated; *red*, upregulated in RO (regenerated). The color intensity is correlated with fold change. *Straight lines* are for direct gene-to-gene interactions, *dashed lines* are for indirect ones

We next sought to examine the overrepresented biological processes of 95 significantly dysregulated genes shared in the regenerated liver and in normal aging, however, in the opposite direction. The biological processes of those genes include signal transduction (21 %), protein metabolism and modification (14 %), developmental process (13 %), transport, and cellular differentiation and proliferation (Fig. 2a). The most significantly enriched biological process categories include ectoderm and mesoderm development, developmental process, small molecule transport, and cell proliferation and differentiation (*p* value <0.05). Consistent with the categories identified by PANTHER, the IPA functional analysis also revealed cellular movement, embryonic development, lipid metabolism, small molecule biochemistry, cellular growth and proliferation, tissue development, and cellular development as significantly enriched functional categories (Fig. 2b).

### Gene interaction network analysis of aging genes reset to young age by regeneration

To obtain a deeper insight into the interaction network and altered pathways, we mapped the 95 aging genes whose expression was set to young age by regeneration to gene networks using the ingenuity knowledge base. These genes were mapped primarily to top networks (Fig. 2c) related to, among others, cellular movement, embryonic development, cellular growth and proliferation, tissue development, lipid metabolism, and small molecule biochemistry. Our gene network analysis indicated important roles of *PAX3*, *E2F1*, *ITGA5*, *MAPK*, and *VCAN* that are interacting with many other genes that we have identified in this study.

### Validation of the results in independent HCC datasets and comparison to calorie restriction studies

As a validation of our results, we re-analyzed three independently performed microarray datasets for human HCC (Wurmbach et al. 2007; Boyault et al. 2007; Mas et al. 2009). The comparison of our rat HCC genes (human orthologous) with the re-analyzed human HCC datasets revealed a significant number of genes in common (*p* value <0.001). Furthermore, we obtained consistent results with two independent studies of DEN-induced HCC in rats (Perez-Carreon et al. 2006; Liu et al. 2009). The functional and gene ontology analyses of all validation datasets revealed a significant number of overrepresented functional categories in common with our results. Of note, cell death, cancer, cellular development, cellular growth and proliferation, organismal development, transport, and cell cycle came up as significantly enriched categories in both the validation datasets and our analyses.

To further validate, we compared our results with independently performed microarray studies for aging and calorie restriction. Most recently, Hong et al. (2010) gathered genes related to aging and calorie restriction (CR) from numerous published studies and from microarray datasets in the GEO repository. We found that a significant number of our aging genes were in common with our analysis results (*p* value <0.05). Furthermore, a significant number of over-represented gene ontology (GO) terms in aging from our analysis were retained in the validation set. For example, immune response, cell adhesion, lipid metabolism, cellular development, nervous system development and function, cell-to-cell signaling, and skeletal and muscular system development and function come up as significantly enriched GO categories in both the validation and our analysis. Hence, the similarities between our results and the independent validation sets argue against random chance accounting for the observed enrichment of these functional categories and pathways.

### Validation of selected differentially expressed genes using real-time RT-PCR

We used quantitative real-time RT-PCR to validate our microarray results for eight randomly selected genes (*E2f5*, *Cep350*, *Nrp1*, *Esm1*, *Cltb*, *Caskin1*, *Wit3*, and *Ube2l6*) (Table 4). A significant correlation existed between the microarray and real-time RT-PCR results (*r* > 0.74, *p* value <0.03). We also validated expression levels of another set of six randomly selected differentially regulated genes (*Pbsn*, *Cdh13*, *Lum*, *Nid2*, *Dcn*, *Slc22a5*) in our earlier study (Colak et al. 2010). In all cases, a high concordance existed between the microarray and real-time RT-PCR results thus demonstrating the reliability of our gene expression measurements.

**Table 4:** Validation of selected differentially expressed genes using real-time RT-PCR

Gene symbol	Description	Hepatoma (DO/NO)		Regenerated (RO/NO)		Normal aging (NO/NY)	
		qRTPCR	Microarray	qRTPCR	Microarray	qRTPCR	Microarray
Caskin1	Cask-interacting protein 1	10.1	3.4	10.3	3.4	−4.9	−3.6
Wit3.0	Wound inducible transcript 3.0	6.8	1.4	4	1.5	−3.9	−1.6
Cltb	Clathrin, light polypeptide (Lcb)	7.4	1.6	4.6	2.5	−1.7	−1.8
Nrp1	Neuropilin 1	1.4	2.5	3.1	2.4	−2.3	−2.5
Cep350	Centrosome-associated protein 350	1.4	3.5	2.5	3.4	−2.3	−8.1
Esm1	Endothelial cell-specific molecule 1	−1.8	−3.4	−1.8	−3.2	10	5.2
Ube2l6	Ubiquitin-conjugating enzyme E2L 6	−2.5	−3.8	−3.3	−2.6	1.8	3
E2f5	E2F transcription factor 5	−1.4	−2.5	−1.8	−1.9	1.4	4.3

## Discussion

The present study sought to investigate if the proliferation in old rat liver can reset the levels of expression of aging-related genes to that observed in young tissue using global gene expression profiling. We developed a rat model of liver regeneration post-hepatectomy (return to quiescence), as well as liver cells undergoing malignant transformation, and compared them to normal liver using a comprehensive microarray of 27,000 publicly available and Celera annotated rat genes. We have found evidence that over 20 % of the aging-related genes had their levels of expression reset to young levels by stimulating proliferation, even in cells that had undergone a limited number of cell cycles and then become quiescent again. Importantly, we identified genes and pathways significantly altered with aging and regeneration that are distinct from cancer.

Aging is a complex process characterized by a gradual and progressive decay of biochemical and physiological functions of most organs. Our data revealed that genes associated with immune and stress response were activated with aging, whereas genes associated with lipid metabolism, cellular growth and maintenance, protein synthesis, and cellular communication were downregulated, consistent with other independent studies, such as gene expression in human retina (Yoshida et al. 2002), gene expression profiling of aging rat liver (Tollet-Egnell et al. 2001), age-associated changes in gene expression in human liver (Thomas et al. 2002), and mouse transcriptome (Hong et al. 2010; Schumacher et al. 2008). The functional pathways were also consistent with previous studies. However, the novelty of our approach is that we identified a novel group of genes and altered pathways involved in regeneration and hepatoma as well as in aging and found genes differentially regulated in both regenerated and hepatomas from old liver, as well as uniquely dysregulated genes in hepatoma and regenerated whose expressions were reset to that seen in normal young. Intriguingly, the comparison of normal aging genes with the significantly dysregulated genes in the regenerated old liver revealed that the expression levels of over 90 % of shared genes had displayed similar levels to those seen in the normal young liver than old liver. This occurred in hepatoma cells that were actively proliferating (Charlton et al. 1999) and, more importantly, also in cells from regenerated liver lobes that had undergone a limited number of cell divisions and then resumed a quiescent state. The gene ontological analysis revealed that the regeneration-specific genes were mainly associated with lipid metabolism, mRNA transcription and regulation, protein modification, protein phosphorylation, cell morphology, cellular development, small molecule biochemistry, and cellular growth and proliferation. Thus, the genes and processes activated or repressed by aging were conversely regulated in the regenerated liver.

CR diet has been shown to have effects on lifespan prolongation, mitochondrial autophagy, cell adaptation to hypoxia, and antineoplastic effects (Li et al. 1997; Cao et al. 2001). Hence, we investigated if there are any similarities between calorie restriction and regeneration-related gene expression changes, as both have demonstrated a reversal of gene expression changes due to aging. Studies on CR-related longevity indicate a number of genes, including silent information regulator 2 (Sir2), Sirtuin 1, forkhead box (FOXO1), that are involved in antineoplastic effects; whereas, FoxM1B transcription factors prevent age-related proliferation defects (Wang et al. 2001; Nasarre et al. 2010; Yamaza et al. 2010). Recently, proteomic profiling was done in aging rat white adipose tissue, and calorie restriction reversed the aging-related protein alteration. Calorie restriction improves the oxidative stress, cellular and energy metabolism, iron storage, and antioxidant affects (Valle et al. 2010). The comparison of CR and our regenerated-related genes that reset the aging genes revealed over-representation of genes related to lipid metabolism, cellular growth and proliferation, transcription, cell adhesion, and response to stimulus (Hong et al. 2010).

Our functional pathway and network analysis of aging genes whose expression was reversed by regeneration indicated alterations in MAPK/ERK and Jun-N-terminal kinase (JNK) pathways. Recent studies have identified the JNK pathway as a regulator of insulin/insulin-like growth factor signaling and influencing growth, metabolism, stress tolerance, and regeneration (Wang et al. 2005, 2003; Karpac et al. 2009). Interestingly, moderate activation of JNK signaling resulted in increased stress tolerance and extended life span in various organisms (Wang et al. 2005, 2003). Moreover, our network analysis also indicated potential important roles of *PAX3*, *VCAN*, *ARRB2*, *ITGA5*, and *NR1H2* that are interacting with many other genes that we have identified in this study. These genes are known to be involved in proliferation, organismal development, and cell adhesion in a wide variety of cell types (Anderson et al. 2001; Matsumura et al. 2003; Conboy et al. 2011). Interestingly, liver X receptor beta (LXR-β) is a member of the nuclear receptor family of transcription factors and encoded by the NR1H2 gene (nuclear receptor subfamily 1, group H, member 2). LXR-β is a close human homologue of daf-12, a regulator of nematode longevity, and is a key regulator of macrophage function, controlling transcriptional programs involved in lipid homeostasis and inflammation (Gerisch et al. 2001; Ly et al. 2000; Gems et al. 1998; Chang et al. 2008). It is therefore possible that these genes were influential in producing the changes in gene expression we have observed as a result of inducing proliferation in old liver and hence warrant further study.

In conclusion, to our knowledge, this is the first study to examine global gene expression patterns in normal aging and regenerated old liver differentiated from hepatic liver and demonstrates that by inducing cells to proliferate, it is possible to reset the gene expression levels in old rat liver to that observed in normal young liver for many of the differentially expressed genes. This occurred in malignant cells that were actively proliferating and also in cells from regenerated liver lobes that had undergone a limited number of cell divisions and then resumed a quiescent state. Intriguingly, the cells do not need to be actively proliferating to maintain the reset state. It is unknown which aspect of cell proliferation is responsible for this effect. However, if this can be identified in future research, then it may prove possible to develop an intervention that can produce an anti-aging effect.
